# Socio-demographic caracteristics and prevalence of risk factors in a hypertensive and diabetics population: a cross-sectional study in primary health care in Brazil

**DOI:** 10.1186/s12889-016-3230-7

**Published:** 2016-07-15

**Authors:** Julio Baldisserotto, Luciane Kopittke, Fulvio Borges Nedel, Silvia Pasa Takeda, Claunara Schilling Mendonça, Sérgio Antonio Sirena, Margarita Silva Diercks, Lena Azeredo de Lima, Belinda Nicolau

**Affiliations:** Social and Preventive Dentistry Department, Faculty of Dentistry, Federal University of Rio Grande do Sul, Rua Ramiro Barcelos 2492, Porto Alegre, CEP 90035-004 Brazil; Education and Research Center in Primary Health Care, CEPAPS and Pos Graduation Programe of Health Technology Assessment of Grupo Hospitalar Conceição, Porto Alegre, Brazil; Health and Science Center, Public Health Department, Federal University of Santa Catarina, Florianópolis, Brazil; Grups de Recerca d’Amèrica i Àfrica Llatines, Unitat de Bioestadística, Facultat de Medicina, Universitat Autònoma de Barcelona GRAAL/UAB, Barcelona, Spain; Education and Research Center in Primary Health Care, Grupo Hospitalar Conceição, Porto Alegre, Brazil; Faculty of Dentistry, Division of Oral Health and Society, McGill University, Montreal, Canadá

**Keywords:** Hypertension, Diabetes mellitus, Primary health care, Service evaluation, Chronic diseases, Self-assessment

## Abstract

**Background:**

Systemic arterial hypertension and diabetes mellitus, and their related morbidity and mortality, are currently the most common public health problems and also a higher burden of disease in Brazil. They represent a real challenge for primary health care. This study describes the methodology and baseline data of an adult population with hypertension and diabetes attending in primary health care.

**Methods:**

It is a cross sectional study which presents data from a longitudinal research. 3784 adults were randomly selected from the registry of a health service in Porto Alegre, Brazil. The eligibility criteria were: confirmed diagnosis of hypertension and/or diabetes, consulted at least once in the prior 3 years and 18 years of age or older. Home data collection consisted of a questionnaire with information on demographic, medical history, life style and socio-economic factors.

**Results:**

A total of 2482 users were interviewed (response rate of 71 %). The median age was 64 (IQR = 55.7) and the majority were women (68 %), and married (52 %). Whereas 66.5 % (CI 95 % 64.5-68.3) of the sample had only hypertension, 6.5 % (CI 95 % 5.5-7.5) had diabetes and 27.1 % (CI 95 % 25.3-28.8) had both diseases. The prevalence of diseases increased with age and with fewer years of study (*p* < 0.05). Subjects with both diseases had significantly more associated comorbidities.

**Conclusions:**

Hypertension and diabetes are more prevalent in older individuals, especially women, and less educated people. People suffering with both chronic conditions simultaneously are more likely to have additional comorbidities.

## Background

Chronic non communicable diseases, especially systemic arterial hypertension and diabetes mellitus, and their related morbidity and mortality, are currently the most common public health problems [1] and also a higher burden of disease in Brazil. They represent a real challenge for primary health care (PHC).

Their prevalence is high in both developed and developing countries [2] and has been on the rise, paralleling the obesity epidemic [3]. Data from from VIGITEL (Vigilance Risk Factors and Protection for Chronic Diseases by Telephonic Inquiry) [4] show that the prevalence of hypertension and diabetes among Brazilian adults aged 35 and older was 24.3 and 11.7 %, respectively. In Porto Alegre city, the rates are higher than the national average and in people aged over 65 the prevalence rises to 54.9 % for hypertension and 19.3 % for diabetes [4].

The causes for the observed increase in the incidence of obesity, and hypertension and diabetes are not completely understood, but changes in lifestyle factors such as higher intakes of empty calorie food, fast food diets and lack of physical activity^3^ are considered the main factors. These diseases, which are also major risk factors for other chronic illnesses [5], are the cause of many hospital admissions [1].

A myriad of studies sustains that the current international consensus of primary health care guided by its essential attributes – access, longitudinality, integrality and coordination - forms the central pillar in fighting chronic non communicable diseases [6, 7]. Currently, the major challenge for primary care is to increase the rate of diagnoses of people with hypertension and diabetes in community settings. The percentage of people with control of these diseases in the medium- and long-term also needs to be increased. This in turn, would reduce the related morbidity and mortality.

Despite the substantial investment and improvement in the Brazilian PHC, the diagnosis and control of hypertension and diabetes are still lower than in countries with similar health care models such as Canada and Cuba [8, 9, 10]. For instance, Brazilian data show that inherent complications such as heart diseases affect 15 % of users, while cerebrovascular accidents (6 %), kidney disease (8 %), peripheral artery disease 6 %, diabetic foot 2 % and retinopathy 5 % [11] account for the other complications.

Specifically in Porto Alegre, capital of Rio Grande do Sul, south of Brazil, 57 % and 44 % of hypertensive and diabetic patients had control of their blood pressure and blood-sugar levels in 2010 [11], according to the Community Health Service (CHS), a primary health service that cares for a 110,000 people in the city. Considering the dimension of the problem described above, the CHS proposed a strategic health policy to be developed along four years aimed at improving access, diagnosis and clinical control of hypertension and diabetes of this population.

The current paper describes, specifically the methodological procedures and the constitution of a baseline related to risk factors and lifestyle, service use and demographic aspects of this diabetic and hypertensive patients enrolled in the CHS.

This research was funded by the Department of Basic Health Care of the Ministry of Health of Brazil, along with the Pan-American Health Organization (PAHO).

## Methods

### Study design

This is a cross sectional study which describes the research methodological aspects and present data of the baseline related to risk factors and lifestyle, service use and demographic aspects of diabetic and hypertensive patients enrrolled in a primary health care service.

This baseline information presented herein constitutes a first stage of a major longitudinal research designed to assess the future impact of a health policy planned to improve hypertension and diabetes diagnosis and control among the users of the Community Health Service (CHS). The ultimate goal of this health policy is to reduce morbidity and mortality rates associated with these conditions through interventions that place a priority on establishing managerial goals (e.g., increase in the % of diagnoses, purchase of equipment, optimize laboratory exams) and changes in care team work processes (e.g., continuing education activities, seminars and workshops, group consultations).

The baseline presented in this paper was concluded prior to the implementation of the previously described series of interventions in the health service.

In order to assess services and programs, epidemiological studies have been recommended. To evaluate a programme or intervention, it is necessary to choose an appropriate design based on the type of decisions that should be taken. For the evaluation study design we used the criteria described by Habicht et al [12].

### Population source and sampling process

The target population of this study lives in the area covered by the Community Health Service and was estimated at 108,000 inhabitants in 2010. It is located in Porto Alegre, the capital of Rio Grande do Sul state, the tenth largest Brazilian city. This health service is public, integrated with the National Health System (SUS) and was chosen due to its structure and its excellence in PHC in Brazil. The research was demanded by the Brazilian Ministry of Health.

Of these, 80,000 adult users are registered in the CHS database system. It is estimated that of the adult population, 20,869 suffer from hypertension (26 %) and 6421 are diabetics (8 %) [13]. To be eligible to enter in the study, the participants had to comply with the following criteria: (i) to be registered in the program called *Hiperdia* (hypertension and diabetes program) and have a confirmed diagnosis of hypertension and diabetes in accord with the International Classification of Diseases (ICD) for hypertension (I10, I11) and/or diabetes (ICDs E10, E11, E12, E13, E14); (ii) to have visited the health service for consultation in the 3 years prior to the study recruitment; (iii) to be 18 years of age or older on March 1, 2009.

A total of 9059 people registered in the hospital database fulfilled these criteria. Out of these 3784 individuals were randomly selected through a simple random process without replacement using a table of random numbers. The sample size was calculated based on the Moivre theorem to permit identification, with an accuracy of 95 % and power of 80 %, with the hypothesis that the intervention plan could achieve a target increase of 10 % in the proportion of users with control of their diabetes (from the current 54 % to 59 %). Using these parameters, the minimum size of the sample was 2646 subjects, to which 105 was added for numerical recovery for loss and another 30 % for greater stability in the multi-variable analysis, resulting in 3784 individuals. We estimated that this number would be sufficient to assess the same change in users with hypertension.

### Data collection

Data collection was conducted by a company specialized in population studies and surveys and included monitoring and supervision by researchers. Subjects were interviewed in their homes by trained interviewers in March and April 2011. Up to four visits were made in an attempt to find the subject home, along with another visit by the supervisor on alternate days to confirm and validate the loss and corresponding reason. Quality control of the visitation process and data collection was implemented through supervision of 20 % of the interviews.

Research manuals (Interviewer Manual, Supervisor Manual and Visitation Form) containing instructions and possible doubts with regards to data collection and home interviews were initially produced.

### Research instrument and variables

The questionnaire used was based on previous studies with added questions and instruments validated in the literature [14]. It was comprised of 86 closed questions, encompassing aspects related to life and health conditions, habits and the use of health services by the interviewee. The information collected was organized into the following blocks: A- demographic; B- lifestyle; C- general health history and D- socio-economic factors, comprising a total of 90 questions.

The Brazilian Association of Research Companies (ABEP) Classification [15] was used to identify the social and economic status of the sample. This criteria system is based on the purchasing power and the level of education of the families, ranging from the highest (Class A) to the lowest (Class E).

Due to a failure in the data collection process, no information was collected on the education of the head of the family, a variable used to construct the social and economic classification. After identifying the error, telephone contact was attempted will all interviewees, though the information return was only 41 %. To overcome this problem, we assessed the possibility of using the education level of the actual interviewee as a replacement for the head of the family in the ABEP classification algorithm, considering that a number of the interviewees are heads of family and have similar education levels. As such, an analysis was made of the concordance between the original ABEP classification (41 % of the cases) and the result from using the education level of the interviewees from this population with the two pieces of information (*n* = 1096) through the kappa index. For the ABEP classification in eight categories, the kappa was 0.90 (CI 95 % 0.89-0.92) and 0.86 (CI 95 % 0.83-0.92) when grouped in three categories A-B; C and D-E.

We used the Brazilian Diet Guide [16] for the classification of diet adaptation, replacing the questions related to physical activity, which was gauged by the International Physical Activity Questionnaire (IPAQ) [17], and to the use of alcohol, measured by the CAGE (acronyms refers to 4 questions: *Cut down, Annoyed by criticism, Guilty e Eye-opener*) [18] questionnaire. As these two variables are dichotomous and replace variables with three categories, the cut points suggested by the Diet Guide were modified for the first quartile, interquartile interval and third quartile of the noted values (up to 28, 29 to 34, over 34), to classify the diet as: inadequate/should be improved, can be improved and healthy. The classification method adopted seems suitable, as the distribution of points is close to a normal curve, practically symmetrical and only very slightly platykurtic.

### Data analysis

As a preliminary step, the distribution of each variable was examined for outliers and obvious errors. Bivariate analyses for numerical variables were conducted using *t* test or ANOVA, when they presented a normal distribution, and the Kruskal-Wallis test for other cases. For the analysis of categorical variables, Fisher’s exact or Pearson’s χ2 tests with corrections by Yates were used. Analyses were calculated with their confidence interval of 95 % (CI).

## Results

Of the total sample of 3784 users randomly selected from the health information system, 2482 individuals were effectively interviewed at home. The major losses were due to the subjects who were not home at the time of the home visits. After four attempts the loss was confirmed. Those who refused to participate represented 1.7 % and 7.4 % were excluded due to incomplete information in their files, change of address or death.

Therefore, our survey response rate was 71 %. It was calculated as the proportion of subjects registered in the database after exclusion of the subjects who were not part of the target population.

Table 1 presents the frequency distribution of social and demographic characteristics of the sample. The median age is 64 years (inter quartile range = 55.7). There is a statistical significance in the difference of gender composition, with women being the majority. More than half (52 %) were married or involved in a stable relationship. Of the total sample, 7 % do not know how to read or only are able to sign their own name. Among people with a formal education (93.0 % - CI: 91.9 - 94.0), more than two thirds have completed primary education and only 5 % have a higher education. Regarding the distribution of social and economic classes according to modified ABEP criteria, the majority of interviewees, 64.3 %, belong to Class C and 0.3 % live in conditions of extreme poverty. Two thirds of the users (66.5 % - CI: 64.5-68.3) studied have hypertension without diabetes, 6,5 % (CI: 5.5-7.5) have diabetes without hypertension and 27,1 % (CI: 25.3-28.8) have both conditions.

**Table 1:** Socio-demographic characteristics according to hypertension and diabetes condition in users of the Community Health Service - Conceição Hospital Group. Porto Alegre, Brazil, 2011

Adults with Chronic Diseases *N* = 2482				
	Hypertension	Diabetes	Hypert + Diab	*p* *
	*n* (%)	*n* (%)	*n* (%)	
	1638 (66,5)	160 (6,5)	684 (27,1)	
	CI 95 % (64.5-68.3)	CI 95 % (5.5-7.5)	CI 95 % (25.3-28.8)	
Gender				0.01
Male	497 (30.3)	67 (41.9)	224 (32.7)	
Female	1141 (69.7)	93 (58.1)	460 (67.3)	
Age				<0.001**
Median ± IQR	64 (54.7)	59 (51.7)	65.5 (58.7)	
Age-Group				<0.001
20 - 39 years old	69 (4.2)	11 (6.9)	9 (1.3)	
40 - 59 years old	574 (35)	71 (44.4)	186 (27.2)	
60 - 74 years old	671 (41)	61 (38.1)	341 (49.8)	
75 - 84 years old	261 (15.9)	14 (8.8)	133 (19.5)	
85 - over	63 (3.8)	3 (1.9)	15 (2.2)	
Economic				0.766
Classes A-B	377 (24.2)	40 (26.3)	164 (25.4)	
Class C	1014 (65.1)	93 (61.2)	406 (62.8)	
Classes D-E	166 (10.7)	19 (12.5)	76 (11.8)	
Able to read/write				0.807
No	53 (3.2)	5 (3.1)	29 (4.2)	
Yes	1520 (92.8)	148 (92.5)	629 (92.0)	
Able to sign name	65 (3.9)	7 (4.4)	26 (3.8)	
Education level				<0.001
< 4th year primary education	274 (18.2)	30 (20.5)	130 (21.0)	
4th year primary education	534 (35.4)	47 (32.2)	257 (41.6)	
Primary Education concluded	362 (24)	25 (17.1)	112 (18.1)	
High School concluded	312 (20.7)	42 (28.8)	105 (17.0)	
Higher education concluded	26 (1.7)	2 (1.4)	14 (2.3)	
Marital Status				0.018
Common Law	847 (51.7)	93 (58.1)	361 (52.8)	
Widow(er)	366 (22.3)	19 (11.9)	164 (24.0)	
Separated	204 (12.5)	21 (13.1)	87 (12.7)	
Single	221 (13.5)	27 (16.9)	72 (10.5)	

*Chi-squared test

** Kruskal-Wallis test

The frequency distribution of hypertension and diabetes associated morbidity is displayed in Table 2. More than seven per cent (CI: 6.4 - 8.54) of the hypertension or diabetes sample needed hospitalization due to complications in the previous year prior to data collection; while 10.6 % (CI: 9.5 - 12.0) of the subjects had a history of acute myocardial infarction, 6.3 % (CI: 5.4 - 7.3) had a cerebrovascular accident, and amputation of a limb due to diabetes occurred in 1.7 % of the diabetics. The diabetic with hypertension group showed the higher percentages in all comorbidities (*p* < 0.005).

**Table 2:** Health conditions of patients with hypertension and diabetes under the care of Community Health Service - Conceição Hospital Group. Porto Alegre, Brazil, 2011

	Hyper	Diabetes	Hyper + Diab	Total	*p* *
	*n* (%)	*n* (%)	*n* (%)	*n* (%)	
	CI 95 %	CI 95 %	CI 95 %	CI 95 %	
Total subjects	1638 (66)	160 (6)	684 (27)	2482 (100)	
Admitted within the last year (missing = 2 %)	101 (6.18)	12 (6.7)	66 (10.15)	179 (7.4)	0.005
	(5.07-7.46)	(4.04-13.05)	(7.94-12.74)	(6.4-8.5)	
Previously suffered Acute Myocardial Infarction (missing < 1 %)	166 (10.16)	7 (2.7)	91 (13.66)	264 (10.6)	0.001
	(8.74-11.73)	(1.78-8.81)	(11.15-16.51)	(9.5-12.0)	
Previously suffered an Encephalic Vascular Accident (missing < 1 %)	102 (6.23)	2 (1.3)	51 (7.66)	155 (6.3)	0.011
	(5.11-7.52)	(0.15-4.44)	(5.75-9.95)	(5.4-7.3)	

*Chi-square test

*Hyper* Hypertension

*Diab* Diabetes

Table 3 displays the lifestyle and behavioral factors of the sample. More than half of the sample reported that they practice regular physical activities, while over 90 % of the interviewees use medication. Regarding smoking and alcohol habits more than half (51 %) have consumed alcohol or tobacco in their life time and of these 5 % consume alcohol excessively. More than half of the sample never smoked. Thirty two per cent (803) are past smokers and 15,1 % (376) are current smokers. The majority felt their health was regular/good (83.4 %) or very good/excellent (5.4 %) and only 11.2 % that is was poor. A higher percentage (16.0 %) of people with diabetes associated to hypertension reported their health as poor. In relation to satisfaction with the health service, almost three-quarters of the sample were very satisfied with the care received.

**Table 3:** Lifestyle and service satisfaction of patients with hypertension and diabetes under the care of Community Health Service - Conceição Hospital Group. Porto Alegre, Brazil, 2011

	Adults with Chronic Diseases *N* = 2482				
	Hyper	Diabetes	Hyper + Diab	OR (95 %)	P*
	*n* (%)	*n* (%)	*n* (%)		
Physical activity					0.594
Yes	840 (51.3)	85 (53.1)	366 (53.5)	1	
No	798 (48.7)	75 (46.9)	318 (46.5)	0.48 (0.46-0.5)	
Medication use					<0.001
Yes	1575 (96.2)	140 (87.5)	612 (91.8)	0.94 (0.93-0.95)	
No	63 (3.8)	20 (12.5)	55 (8.2)	1	
Excessive alcohol use					0.188
Yes	84 (5.1)	12 (7.5)	28 (4.1)	0.51 (0.48-0.53)	
No	1554 (94.9)	148 (92.5)	656 (95.9)	1	
Smoking					0.001
Never	873 (53.3)	77 (48.1)	353 (51.6)	0.53 (0.51-0.64)	
Stopped	500 (30.5)	50 (31.2)	253 (37.0)	0.32 (0.30-0.34)	
Ever	265 (16.2)	33 (20.6)	78 (11.4)	0.15 (0.14-0.17)	
Health self-evaluation					<0.001
Poor	156 (9.5)	11 (6.9)	109 (16.0)	0.11 (0.10-0.12)	
Regular/Good	1384 (84.6)	138 (86.8)	542 (79.7)	0.83 (0.82-0.85)	
VG/Excellent	95 (5.8)	10 (6.3)	29 (4.3)	0.05 (0.04-0.64)	
Service use					0.888
Unsatisfied	56 (3.4)	6 (3.7)	29 (4.2)	0.05	
Regular/Satisf	327 (20.0)	34 (21.3)	139 (20.4)	0.11	
VS/Excellent	1253 (76.6)	120 (75.0)	515 (75.4)	0.83	

* Chi-squared test, *VG* Very Good, *VS* Very Satisfied

According to a study derived from this research and recently published, it was found that eating habits among diabetic and hypertensive subjects were deleterious: low intake of fibers and high intake of sugar, salt and fat, being the diet worse for those suffering from hypertension only [19].

## Discussion

This paper aims to describe the methodological procedures and baseline results of a research project that was set up to assess the future impact of a strategic health policy on the quality of care offered at the Community Health Service, a federal public institution integrated with the National Health System (SUS) in Porto Alegre, Brazil. It is important to highlight that this research, which was entirely planned, executed and analyzed by a health service team from the National Health System, is an uncommon practice in the Brazilian PHC. Based on epidemiological data and evaluations generated locally, the team noticed the need to enhance the quality of the services offered to hypertension and diabetes users. Thus, a health policy including a series of managerial interventions were proposed to re-organize and re-structure the service. Our ultimate goal was to set a monitoring study model to evaluate the impact of a strategic plan on the diagnosis and clinical control of hypertension and diabetes over an extended period of time.

Among social and economic determinants investigated, our baseline results show that our sample has a similar socio-demographic profile as reported in other studies including the National Health Survey [20]. The majority of people with hypertension and diabetes were above 40 years of age and these conditions increase significantly with age. These patients tend to have a lower education and are predominantly women.

Concerning smoking and alcohol use, both considered important risk factors in the development of chronic diseases, 15 % of the sample responded that they were active smokers and 5 % used alcohol excessively. Considering that the sample population is comprised of people with hypertension and diabetes and that they visit health services quite regularly, a lower rate of tobacco use was expected. These numbers may indicate a need to review certain educational approaches conducted in this health service.

Another factor analyzed in the study concerns the practice of daily physical activity. There is a plethora of evidence showing the positive effects of physical activity on both prevention and non-pharmacological treatment of patients with chronic illnesses [21]. However, more than half (52 %) of our sample responded that they did not routinely practice physical activity. This finding is similar to those reported in a study by Newson et al [22]. They show that Canadians aged 50 or older made only modest changes in their behavior after being diagnosed with chronic conditions, after 12 years of follow-up. The authors argue that age is an important barrier to implementing physical activity among those suffering from chronic illnesses. Furthermore, physical inactivity increases with age among different populations including Brazilians [23]. Sixty-three percent of our study sample was 60 years old and over, and this might explain why the majority of our patients did not exercise. More importantly, these results pose a big challenge to health care teams in terms of developing strategies to improve compliance with exercise recommendations among this population.

According to Alderman [24], the majority of hypertension and diabetes cases and their associated risk factors are more appropriately managed with the support of primary health care services. That is, the quality of care for these conditions prevents hospitalization and death through heart and cerebrovascular complications. Indeed, data on hospital admissions relative to primary care sensitive conditions confirm that greater involvement of PHC teams is associated to sharp drops [25], and lower rates of admissions, including those resulting from chronic diseases [26]. The importance of PHC services and their action related to a range of risk factors linked to people’s lifestyle and environment has been documented in the literature [25, 26].

In Brazil, the challenges of controlling and preventing hypertension and its complications are under the responsibility of Basic Health Care teams. The multi-professional teams follow work processes based on community involvement, facilitating comprehension of the health-disease process and its determinants in the individual’s lifestyle. However, this approach has been proved not to be completely successful. Results from a Canadian longitudinal study indicate that changes in lifestyle were modest, even after chronic illnesses were diagnosed. There was no significant reduction in alcohol consumption, nor improvements in physical activity or eating habits in this population after having being diagnosed with some form of chronic illness [22]. Similarly, Brazilian elderly suffering from hypertension reported that harmful health habits (physical inactivity, inadequate diet, use of alcohol) persist throughout their disease with the exception of tobacco use [27]. Similar results were found in our study in which, among risk factors, there was an improvement in the number of people who stopped smoking.

A systematic review addressing the effectiveness of health programs in improving outcomes (related to physical or mental health, service utilization, medication adherence, and others) of patients with multiple comorbidities showed that the majority of the changes were related to the organization of primary health care. In general, the most positive results were those focused on risk factors of specific population groups [28]. Studies have shown that even in countries with good basic health care coverage, there is still great difficulty in diagnosing people with hypertension. That is the case of Canada where treatment rates are high in those who are diagnosed (95 %), but 17 % of people with hypertension remain undiagnosed [10].

Our results and the scientific literature provide evidence that it is necessary to find effective strategies to bolster self-care and reinforce the autonomy of individuals facing hypertension and diabetes.

We are already using the data from the baseline wave of this study to implement strategies addressing the issues discussed above in our community health centers in a catchment area of 120,000 people. For example, we implemented protocols and a continuing education program for health professionals. Moreover, we implemented an evidence based model of care centered on the patient and on the community with the goal of encouraging lifestyle changes. As such, we have leveraged the role of PHC in acting to preventing and controlling the leading risk factors of chronic diseases. Another challenge lies in increasing the rate of diagnosis of hypertension and diabetes, while also increasing the percentage of people with clinical control of these diseases.

One of the possible limitations of this investigation is related to the initial loss of subjects. Out of 3784 patients randomly selected from the registrations in the Community Health Service Information System, we were able to trace 2482. The majority of losses were due to problems with the subjects’ addresses which were incomplete or incorrect. This provides an indication that the medical files registrations made at our 12 community health centers are not optimal. However, these results can be used to improve the quality of the service. Moreover, out of the 3784 of all the addresses found and visited only 1.7 % refused to participate in the study, which is a very low refusal rate.

The study design used in the main investigation may also be seen as a limitation. However, it is not always possible, ethical and necessary to have a control group when assessing the impact of managerial, educational and health care measures that involve the entire local health system [29]. Moreover, the design of this study was based on elements from population health interventions and/or programs that include assessments of the offer of care, both human and technological, as proposed by Santos and Victora [29]. The use and cover of the offer is also evaluated, along with their impact on the health of the population. Furthermore, this is one of the first Brazilian studies conducted among patients with hypertension and diabetes receiving care from a primary health care service in the south of Brazil. Despite the significant scientific production regarding these leading chronic diseases and their risk factors in Brazil, there are still very few studies evaluating primary health care [30]. Studies such as ours are important because they constitute a key non-experimental outline to expand knowledge on the etiopathogenesis of diseases, in our case hypertension and diabetes, and allow for the planning of strategies to face, prevent and control diseases, and enhance diagnostic criteria and treatment. Currently, ELSA-Brasil [31] is the largest and most comprehensive study on adult health underway in the country. However, this study involves employees from public universities whereas ours encompasses the primary health care network based in community settings and may serve as a reference for future investigations in this area.

## Conclusions

The prevalence of hypertension and diabetes increases with age and gender, being women more affected. The educational level is also associated with the prevalence of these chronic conditions and people having both are more likely to have more comorbidities. This study, besides proving appropriated to the aims of the proposal, will also allow that the impact of different actions and strategies to be implemented in the health service would be assessed through subsequent data collection.

## Abbreviations

ABEP, Brazilian Association of Research Companies; CAGE, cut down, annoyed by criticism, guilty e eye-opener; CEPAPS, Education and Research Center in Primary Health Care; CHS, community health service; IPAQ, International Physical Activity Questionnaire; PAHO, Pan American Health Organization; PHC, primary health care; SUS, unified health system
